# The first stop of transit migrant students on the road to Europe and America: The Turkish education system

**DOI:** 10.3389/fpsyg.2022.1029555

**Published:** 2022-11-14

**Authors:** Mustafa İçen, Şahin Oruç, Mustafa Şeker

**Affiliations:** Faculty of Education, Yildiz Technical University, Istanbul, Turkey

**Keywords:** education, multicultural education, refugee student, transit migrant, Turkish education system

## Abstract

There have been forced migrations from one country to another due to wars, conflicts, and various reasons within and among countries. Turkey is a transit point from Asia to Europe. Accordingly, this study sheds light on experiences the refugee students have in the Turkish Education System. Therefore, this study is meant to be based on a qualitative approach as a case study method. The data were obtained through a semi-structured interview and a focus group interview. The information was gathered from nine students-three Syrians, two Iranians, two Iraqis, and two Afghans-as well as five teachers. According to the results, refugee students had difficulty learning and using a new language, adapting to a new country and environment, and getting used to new friends they made and teachers they were taught by. The results also indicate that students were concerned about their inability to travel to the United States and European countries.

## Introduction

Turkey, which signed the 1951 Geneva Convention, approved of refugees’ status, considering the geographical limitations. Although Turkey does not see itself as a country of immigration, many asylum-seekers and refugees have arrived in Turkey since 1980 (Içduygu, 2016). Notably, since the civil war in Syria, Syrian refugees have begun to migrate to Turkey. According to the Bureau of Citizenship and Immigration Services in Turkey, it is seen that many asylum-seekers from Afghanistan, as well as from Syria, Iraq, and Iran, have applied for refugee status (Soykan, 2010).

Refugee students face various problems, one of which is education, for multiple reasons. According to the data of the United Nations High Commissioner for Refugees, by the end of 2019, 79.5 million individuals had been forcibly displaced worldwide for various reasons. 4 million refugees live in Turkey. Every year, the number of people who leave their homeland rises (UNHCR, 2017,^2019^). Stated that Turkey was the central place of residence for refugees in this context. According to a report published in 2018, Turkey provides shelter for 3.6 million refugees compared to other countries and hosts them (UNHCR, 2018,^2019^; UNHCR Mid-year trends, 2018).

The same report (UNHCR, 2017,^2018^) informs us that Turkey hosts 38.700 refugees from Iraq, 8,800 from Iran, 6.400 from Afghanistan, and approximately 3.6 million Syrians. This indicates that Turkey makes a considerable effort on in regard to refugee hosting. Some cities have been called pilot cities for asylum-seekers and refugees in Turkey. Additionally, public schools were allocated in each town to serve for their educational needs. These schools are the centers that offer refugee and asylum-seeker students many opportunities to prepare them for a new place, increase their well-being, improve their language skills, and help them to thrive like other students.

Considering the number of refugees and asylum-seeker students, the gathered data on this subject are insufficient. However, the possible problems that these students may encounter should be identified and taken into account. Students who are asylum-seekers, refugees, or migrants are never responsible for anything they face. Tilley (2008) emphasized the importance of the rights of students who are asylum-seekers or refugees. These students should feel that they are also children, as well as the right of education they naturally have. In this regard, schools can play an essential role in helping refugee and asylum-seeker students make friends with other students (Uptin et al., 2013; UNICEF, 2014).

Many studies indicate that refugee students face difficulties in their new schools along with those in their new lives (Doner et al., 2013; Arabaci et al., 2014; Emin, 2016; Erdem, 2017; Imamoglu and Caliskan, 2017; Kardes and Akman, 2018; Solak and Celik, 2018; Zayimoğlu-Öztürk, 2018; Alkan and Icen, 2019; Arar et al., 2019; Ozenc and Saat, 2020). Considering the literature in Turkey, it is observed that there are some studies based on teachers’ views on the difficulties that Syrian students face (Arabaci et al., 2014; Solak and Celik, 2018); the problems Syrian students face in Turkey (Doner et al., 2013); experiences the teachers have so far had with refugee students (Erdem, 2017); and educational support is given to refugee students (Emin, 2016; Imamoglu and Caliskan, 2017; Kardes and Akman, 2018, and Zayimoğlu-Öztürk, 2018; Çalışkan, 2020; Gümüş et al., 2020). At the same time, some other studies have been conducted by European countries with similar results (Jeffery and Murison, 2011; Arnot and Pinson, 2015; Dryden-Peterson, 2015; Mixed Migration Platform, 2017).

Given the growth in the number of asylum-seekers and refugees in Turkey, the study focuses on what refugee and asylum-seeker students face in the Turkish education system before they proceed with the European countries and the United States. As stated in UNCHR, Turkey is one of the countries that host the most immigrants. Consequently, there are many studies on immigrants in Turkey, but these studies are lacking in terms of students who want to immigrate to Western countries and their reasons. It is expected that this research will contribute to this deficiency. Thus, the present study aims to extend the knowledge and awareness of students who are “transit migrants.” On the one hand, it aims to add to the literature. In addition, it is meant to contribute to Turkey’s data in order for refugee and asylum-seeker students to be supported as required. In this way, it will be possible to hear what those students say and find out what they need the most.

In addition to the studies on transit migrant, refugee, and asylum-seeker students, this study also reflects the views of students and teachers. The study can help policymakers or teachers realize what they lack in teaching refugee or asylum-seeker students. At the same time, the findings of this study can help policymakers in Turkey, European countries, and the United States design teaching models for refugee and asylum-seeker students and support multicultural projects.

## Methodology

This study was generated on a qualitative approach to understand what asylum-seekers, refugees, and refugee students experience in the Turkish education system. For this reason, an inductive approach was used to establish a relationship between the experiences of asylum-seekers, refugees, and refugee students in the Turkish education system and the study results. As suggested by Bryman (2012), epistemological and ontological positions determine the process of collecting data and interpreting the obtained data throughout the study. In this sense, the interpretive epistemological stance has enabled us to get helpful information from asylum-seekers, refugees, and refugee students, as well as teachers. At the same time, the constructive ontological approach has supported our interpretation. For this purpose, the case study method has been selected.

A case study is a research method that “investigates a current situation in a real-life environment, especially when the relationship between the situation and the environment is not fully seen” (Yin, 1994). This method is also used to investigate an individual, a group of people, or an event (Creswell, 2013). This research design was used to look into the lives of refugee and asylum-seeker students at a Turkish primary school based on what the students, parents, and teachers thought.

### Study group

In order to select convenient participants for this study, the purposive sampling method was used, which “sampled the situations/participants strategically and thus demonstrated the convenience of the sampled ones to the research questions” (Bryman, 2012). According to this method, the middle school where the refugee and asylum-seeker students were studying was intentionally chosen by the Istanbul Provincial Directorate of National Education. Besides, nine middle school students and five teachers who have been teaching these students in this school were selected in the same way According to Silverman (2000), gathering information from various participant groups for a situation promotes validity. Before the data collection process started, the participants were informed that their names would be coded in aliases in the study report. After the necessary permission was obtained, interviews with the volunteers were initiated. In order to select convenient participants for this study, the purposive sampling method was used, which “sampled the situations/participants strategically and thus demonstrated the convenience of the sampled ones to the research questions” (Bryman, 2012). According to this method, the middle school where the refugee and asylum-seeker students were studying was chosen purposefully by the Istanbul Provincial Directorate of National Education. Besides, nine middle school students and five teachers who have been teaching these students were selected in the same way by using purposeful sampling. According to Silverman (2000), gathering information from various participant groups for a situation promotes validity. Before the data collection process started, the participants were informed that their names would be coded in aliases in the study report. After the necessary permission was obtained, interviews with the volunteers were initiated. Ethics committee approval of our research was obtained from Yildiz Technical University (Turkey). Consent of the participants in the study was also obtained.

### Data collection

Data were collected using a semi-structured interview technique. Mason (2002) and Ahrens et al. (2016) stated that semi-structured interviews made it possible for the experiences of the participants to be heard interactively. Two separate interview forms were prepared for each group of students and teachers. Interview forms asked mostly about how the students managed to adapt to school and their peers, how difficult it was to learn, and how they interacted.

### Validity and reliability

In order to support reliability, pilot interviews were conducted with only two students as well as two teachers specified for the pilot project. During the pilot project process, it turned out that some of the students needed additional help to understand what was asked for, in some cases due to insufficiency in language. Therefore, pilot interviews continued in Turkish, Arabic, and English. During these interviews, the use of more than one language helped to detail what was said.

The reliability of this study was backed by the pilot project of data collection tools (Silverman, 1993, 2005). In addition, during the pilot project process, whether any situations needed to be clarified or paid attention to. This pilot project process has shown consistency between the interpretation of the questions by the participant and those meant to be expressed. Additionally, each participant was given their interview transcript and interpretation to check the correctness of their opinions and presentations due to the language problem. Participant control supported participant reliability by providing the consent of the participants. This reliability type has been suggested in studies by Lincoln and Guba (1985) and Cresswell and Miller (2000) to support the credibility and validity of the data. This indicates that the study results will be able to represent the views of other asylum-seekers, refugees, and refugee students in Turkey. In addition, the use of purposeful sampling methods supported external validity in this study (Creswell, 2003).

The interviews focused on the opinions of students and teachers considering the experiences of asylum-seekers, refugees, and refugee students. The experiences of asylum-seekers, refugees, and refugee students have been verified based on the actual views of the participants. Verma and Malick (1999) argued that this situation supports internal validity.

### Data analysis

Thomas (2006) stated that the inductive approach is a systematic way of analyzing qualitative data that allows the researcher to shorten the detailed data into summary form and establish a link between the aim of the study and its findings. According to this point of view, the data in this study were analyzed using the inductive analysis approach, along with? The same ideas presented in the same groups. The thematic analysis method rules were applied to understand the participants’ views based on this approach. Themes and sub-themes were formed in accordance with the thematic analysis method, and the data were grouped under these themes, as suggested by Bryman (2012). Braun and Clarke (2006) divided themes into two: semantic and latent.

Similarly, Braun and Clarke (2006) stated that data in semantic themes are defined in their explicit or apparent meanings, so the analyst understands that the participant does not mean anything else. In this context, this study paid attention to defining the themes during the data analysis process and grouping similar and different views under the correct theme. In addition to this, the real meanings of the words the participants said were also considered.

## Findings

This study aims to determine what asylum-seeker, refugee, and refugee students experience in the Turkish education system. The findings were gathered under three main themes according to the purpose of the research. The themes are as follows:

1. Lack of language Proficiency.
2. Difficulties Caused by Migration.
3. The Adaptation Process and Ongoing Hesitations.

### Lack of language proficiency

The data indicate that asylum-seekers, refugees, and refugee students have difficulties learning and using Turkish, which is a new language for them. Teachers stated that students were not willing to learn Turkish at first. Migrant students do not prioritize learning Turkish because they refuse to stay in Turkey. In other words, it is observed that they believe they would have to stay in Turkey if they learned Turkish. For example, a Syrian student, Aziz, expressed his feelings: *“I did not have to learn Turkish. I was going to go to America soon. […] I started to learn Turkish, and I could not go to America.” A*n Iraqi student, Hassan, stated similar things: “*I did not think I had time to learn Turkish because I was here to go to Europe. I always thought I would be in Germany soon. […] I learned Turkish as much as I needed in social life. Istanbul is a vast city, like a country alone. I have to learn Turkish.*”

Based on the data, language barriers can cause migrant students to fail in communication with Turkish students and teachers. For this reason, successful communication may not have been established among them. Thus, this affected their interactions along with their future learning processes. It is seen that the lack of a common language causes migrant students to have problems in their school lives.

It has been revealed that migrant students sometimes do not understand why Turkish students try to make friends with them. For example, a migrant student, Forouzan, said:

*“When I first came to the class, Turkish friends asked me why we had come to Turkey… That day I thought that they did not like me and did not want me in the class. […] Now, I know my friends love me. It was my misunderstanding […].”*

Teachers stated that although they try to get migrant students to interact, they behave more doubtfully. It is observed that there are misunderstandings due to a lack of language proficiency.

One of the teachers, Davut, expressed his feelings: *“I can speak neither Persian, Arabic, nor English. So I could not communicate with them efficiently. But I would love to be able to talk to them comfortably and to convey my good feelings and thoughts to them.”* Migrant students supported this statement in the focus group interviews: *“Not all Turkish teachers in our school speak Persian or English, so we had to learn Turkish.”* These sentences showed that both teachers and students had communication difficulties. An Iranian student, Maya said: *“I decided to learn Turkish too late because I think it was too late for me to understand the truth that I had to stay here.”*

The data obtained from the teachers revealed that the lack of language proficiency is the biggest problem in the classroom environment. Teachers admitted to not being able to help students learn efficiently in the classroom due to the language problem. According to the findings, teachers become unhappy because they cannot support students’ learning. On the other hand, with the delay in their visit to European countries and the United States, migrant students have realized that they will stay longer in Turkey. Later, it was made clear that they had already started learning Turkish. However, it is understood that this delay in students’ learning Turkish also causes them to fall behind other students.

The teachers emphasized that they had received no training to teach international students. Teachers also stated that they had difficulties responding to students from different countries, motivating them, and managing the teaching process. Ahmet, a teacher, expressed his efforts to solve the problem as:

“*[…] the age of the students, their reasons to migrate to Turkey, and their lack of language proficiency had loaded me with stress and anxiety/ […] I taught them as I did the Turkish students, but I also cared about the differences in between.*”

Ayşe, a teacher teaching international students, also mentioned her efforts in solving the problem:

“*[…]. I acted with the understanding that the teacher is a teacher all over the world, and first of all, I tried to appeal to their feelings. I helped my foreign students feel valued, and I got in return for my efforts.”*

The data from teachers indicated that they make great efforts to motivate migrant students and improve their learning.

The obtained data show that asylum-seekers, refugees, and refugee students have experienced a lack of communication in Turkey due to beliefs that they will not stay long in Turkey, compliance problems with their peers and schools, and language. These prevented them from taking active part in learning activities.

### Difficulties caused by migration

Findings obtained from interviews with all participants revealed that Syrian and Iranian students have difficulty adapting to their schools, teachers, and peers. One of the reasons these students have difficulty is their denial of accepting Turkey as their home country. According to the findings, Syrian and Iranian students did not accept Turkey as their home country because these students were aware of themselves being transit migrants. On the other hand, Iraqi and Afghan students find Turkey livable. Still, they want to go to developed European countries and the United States since they have more economic opportunities.

This led to the feeling that they did not belong to this country. For example, Şamil, a teacher, said: *“What I noticed when I was talking to […] Nejad (Iranian) is that he did not want to stay here for a long time, he wanted America to be his real homeland.”* According to this teacher, when Nejad first arrived in Turkey, he thought he would go to the USA shortly afterward. Davut, another teacher, said: *“I think their parents told them that they would temporarily stay in Turkey.”*

Nejad (Iranian) said, *“We were dreaming that we would go to America soon. I do not know why, but we waited for 1.5 years. We will do whatever we want in America. America is the land of freedom and opportunity.”* This Iranian student’s view indicates that he is not happy to live in Turkey longer than they expected.

Student Jabari (Iraqi) said: “*My mother told me we are here for a holiday. […] The USA would be our real country. My mother said we had been waiting for the tickets for our flight to the USA.*” Student Gazzel (Syrian) agreed with the Iraqi student Jabari: *“America is like a fairground, I will go to department stores, big libraries, and enjoy everything.*” These findings indicate that students believe they will be happier in the USA than in their own countries and Turkey. In other words, students had difficulty adapting to life and school in Turkey because they believed that the United States would be their new home and that they would be safe there.

Nazanin (an Afghan student), *“Turkey is just a place where we are forced to stay. Our Country is the USA.”* As the statement goes, the other students also stated that the country they want to reach is the United States and that their real friends are there. The teachers stated that students hope to go to the United States soon, so they do not try to get used to living in Turkey, the conditions in Turkey, and their friends at school. Even if it has been about 2 years since they came to Turkey, students still seem indecisive and doubtful. However, according to the findings, it can be said that they have started to get used to living in Turkey with Turkish people during this period. Students said Turkey could not be their home country, but it could be their second country. Iraqi and Afghan students think more differently than Syrian and Iranian students. According to them, Turkey can be their homeland, but if any opportunities were given, they would prefer to live in European countries or the United States. The findings obtained from the teachers indicated that students feel more comfortable after gaining more experience in Turkey, Turkish culture, friendships, and life in Turkey.

On the other hand, the data indicate that teachers tell students that they will soon be in the United States. Ayşe, a teacher, said: *“I did not want my students to be disappointed because he thought he would go to the USA at any time. I sometimes told him that he was our guest, and he would go to the USA.”* This shows that teachers do not want their students to have more problems leaving Turkey.

These students were too young to take on the responsibilities of migration. The findings make it clear that students were unhappy when they migrated to Turkey. Student Jabari (Iraqi) said, *“When I first came to the class, they (my friends) were looking at my face in confusion. I felt with all my heart that they did not want me. I did not come to school the next day.”* This student stated that he felt guilty when he was with his peers because he thought he was different from other students. Sadly, other students also mentioned similar experiences they had. This uncertainty about the new location and new friends caused migrant students to feel anxious. Yasemen, another Syrian student, stated that she was welcomed warmly and helped by her new friends. The teachers stated that Turkish students were informed about migration and how to behave when meeting new migrant students.

According to the findings, these students have had severe difficulties for several months. The data gathered point out that one of the causes of these difficulties is that Turkish students despise asylum-seekers, refugees, and refugee students. The migrant students also stated that they realized that they were senseless after getting used to the classroom. Another reason is communication problems between migrant students and Turkish students and teachers. Salih, a teacher, said: “*I did not know the language they spoke. It wasn’t easy to interact with. […] Yes, for a few months, I could not motivate them as I wished. Thus, I could not help the students to communicate with each other, either.”* Students also stated that they could not communicate with each other due to the lack of language proficiency, and this situation made them feel alone and nervous.

Another teacher, Hicran, said: *“I can say that all asylum-seekers and refugees were worried. They had different cultures, and this situation was understandable so I was able to understand their anxiety.”* According to the findings, it can be said that the students’ inability to adapt to the new culture and new rules raises their hesitancy and anxiety. Besides, the data obtained from the students show that they have negative feelings. After all, they think their classmates will make fun of them because they cannot speak Turkish and are unaware of their new living conditions. According to the data collected from teachers, students could not find what they owned in their home countries or what they will have in the US in Turkey. It has also been revealed that students could not socialize because they thought they were from different countries and could not interact with their classmates.

One of the students said: *“There were many times that I felt alone in Turkey. […] Although we have some similarities to Turkish culture, one gets used to it in time.”* This indicates that the students began to get used to living in a new place after a while because they noticed similarities among themselves and their peers and the culture in Turkey.

### The adaptation process and ongoing hesitations

Data show that these participants expect to go to European countries or the United States as soon as possible. They thought they might experience some difficulties in their migration because of the political problems between Turkey and the United States. The students also stated that they do not want US President Donald Trump to be elected again due to his anti-migrant political rhetoric. This stirs anxiety and stresses them about living in Turkey and not moving to the United States. Similarly, it is observed that students follow the statements and agenda of the US president to any extent they can.

Ahmet, a teacher, said he decided that students would not accept to live in Turkey and study in Turkish schools from what he could observe their behavior and attitudes. When asked about his decision, he gave examples of the statements by Iranian and Syrian students statements: *“We do not belong to this school here. We will leave here soon. We do not have to be friends with the students here.”* Other teachers, Salih, Davut, Ayşe, and Hicran, also stated that whenever they tried to motivate students, they repeatedly realized that these students isolated themselves and refused to participate in class activities with their peers.

A student’s statement supported the teachers’ views about Turkey to accept to get used to new peers. An Iranian student, Maya gave an example in the interview:

*“I found Mustafa and Ali very friendly because they tried to help me every day. Although we did not understand each other, we tried to communicate and understand each other. Nevertheless, I did not see them as my real friends because we are going to move to America soon.”*

Data show that students believe that they cannot go to the US from Turkey when they interact with others. Besides, another point of data obtained from the students is that they cannot adapt to life in Turkey because they believe they will not stay long in Turkey.

For this reason, it was unraveled that the students isolated themselves from their environment. One of the teachers (Hicran) said, *“They expected to go earlier, but time has passed, and now they know that they might stay here longer than they thought.”* Based on the data, it can be said that the students started to adapt to their new lives after a few months passed.

As the data show, one of the reasons students have not been able to get used to the new conditions is their parents. In other words, according to the data, parents, consciously or unconsciously, can be said to have caused their children not to adapt to their new lives. For example, a Syrian student (Aziz) stated: *“My family insistently warned me every week that we would be leaving here soon […] so I did not prefer to spend my time with my peers. I did not talk to my teacher. This is not my real city.”* It was revealed that the parents did not want their children to make friends with their Turkish peers because they thought they would go to the US. It is understood that this is the way parents find in the course of immigration to the USA, so their children do not feel sad and depressed. Additionally, it has turned out that parents of the students convince their children that they do not belong to Turkey. This reveals that parents do not want to stay more in Turkey, and they do not want their children to feel that they belong to Turkey.

## Discussion, conclusion, and suggestions

This study aimed to examine and understand what asylum-seekers, refugees, and refugee students experience in the Turkish education system. For this purpose, this study was designed as a case study in compliance with the qualitative approach. A semi-structured interview form prepared for each participant group was used in collecting the data. Thematic analysis methods based on an inductive approach were used to analyze the data. The results were grouped under the themes obtained for this study.

The language problem has been observed as a significant problem for both parties. Therefore, both parties were seen to have difficulties interacting in the learning and communication processes. In line with the results obtained, it can be said that a lack of language proficiency causes reluctance to participate in learning activities and an inability to socialize. School is a vital atmosphere where students have the opportunity to make new friends with their peers and learn more about the new culture while communicating with each other. Therefore, this study aims to gain insight into the experiences of students’ experiences in a school. Besides, school is an important place where educational support is expected for newcomers.

Additionally, school is a significant place that protects students from problems outside of school and ensures that they do not fall behind their peers. It should not be forgotten that the students subjected to research are too young to take on migration responsibilities. However, these students have had to change their lives and adapt to new conditions. The study results indicate that Turkish students were informed about migrant students and approached them in a friendly way. It can be said that there is no effective interaction between migrant students and their teachers. It has been seen that the lack of interaction causes migrant students to misunderstand their friends and therefore isolate themselves.

The study results reveal that asylum-seekers, refugees, and refugee students experience problems due to a language sufficiency, difficulties caused by migration, the adaptation process to the country, and ongoing hesitations. Considering the adaptation process to the country and ongoing hesitations, it has been witnessed that migrant students have difficulties adapting to the new school, new teachers, and new peers as a result of not accepting Turkey as their homeland. Additionally, it can also be said that the delays in the immigration of migrant students and their families to European countries and the United States worry them more. Moreover, it has also been found that migrant students who participated in the study prefer to go to European countries, especially to the United States, rather than Turkey. The data indicate that migrant students believe they will be much happier in the USA than in Turkey and their own country. Also, as per the results obtained, these students can be said to have started to get used to living in Turkish culture due to delays in their immigration to the countries they have been wanting to for various reasons. This result indicates that these students also learned more about Turkey, Turkish culture, and living with their Turkish peers. The results of this study show similarity to many studies conducted with Syrian participants about the language difficulties and adaptation problems (Doner et al., 2013; Arabaci et al., 2014; Emin, 2016; Erdem, 2017; Imamoglu and Caliskan, 2017; Kardes and Akman, 2018; Solak and Celik, 2018; Zayimoğlu-Öztürk, 2018; Alkan and Icen, 2019; Koç, 2020a). Similarly, the results of this study are seen to have matched the results of the projects conducted by European countries (Arnot and Pinson, 2015; Dryden-Peterson, 2015; Içduygu, 2016; Mixed Migration Platform, 2017; Basar et al., 2018; Dogutas, 2018; Koç, 2020b). This indicates that a lack of language proficiency and adaptation to a new place are common problems for all refugees and migrants.

It should be noted that this study was conducted with a participant group consisting of students and teachers, and another study could be done with different participant groups. Furthermore, another study can be conducted using both qualitative and quantitative methods.

The results of this research show that the participating students have similar views about their experiences. It can be said that there is a need for an inclusive “citizenship education” that will support intercultural understanding and cooperation in education systems and schools for students who are refugees and asylum-seekers. Therefore, it should be noted that more studies could focus on this issue.

## Data availability statement

The raw data supporting the conclusions of this article will be made available by the authors, without undue reservation.

## Ethics statement

The studies involving human participants were reviewed and approved by Turkey/Istanbul Yıldız Technical University Ethics Committee. Written informed consent to participate in this study was provided by the participants' legal guardian/next of kin.

## Author contributions

Mİ performed the initial analyses and wrote the manuscript. ŞO and MŞ assisted in the data collection and data analysis. All authors revised and approved the submitted version of the manuscript. All authors contributed to the article and approved the submitted version.

## Conflict of interest

The authors declare that the research was conducted in the absence of any commercial or financial relationships that could be construed as a potential conflict of interest.

## Publisher’s note

All claims expressed in this article are solely those of the authors and do not necessarily represent those of their affiliated organizations, or those of the publisher, the editors and the reviewers. Any product that may be evaluated in this article, or claim that may be made by its manufacturer, is not guaranteed or endorsed by the publisher.
